# Barriers to pandemic influenza vaccination and uptake of seasonal influenza vaccine in the post-pandemic season in Germany

**DOI:** 10.1186/1471-2458-12-938

**Published:** 2012-10-31

**Authors:** Merle M Böhmer, Dietmar Walter, Gerhard Falkenhorst, Stephan Müters, Gérard Krause, Ole Wichmann

**Affiliations:** 1 Immunization Unit, Robert Koch Institute, Berlin, Germany; 2 Department for Infectious Disease Epidemiology, Robert Koch Institute, Berlin, Germany; 3 Department of Epidemiology and Health Reporting, Robert Koch Institute, Berlin, Germany; 4 Charité – University Medicine Berlin, Berlin, Germany; 5 Department for Infectious Disease Epidemiology, Immunization Unit, Robert Koch Institute, DGZ-Ring 1, Berlin, 13086, Germany

**Keywords:** Vaccination, Influenza, Coverage, Pandemic, Germany

## Abstract

**Background:**

In Germany, annual vaccination against seasonal influenza is recommended for certain target groups (e.g. persons aged ≥60 years, chronically ill persons, healthcare workers (HCW)). In season 2009/10, vaccination against pandemic influenza A(H1N1)pdm09, which was controversially discussed in the public, was recommended for the whole population. The objectives of this study were to assess vaccination coverage for seasonal (seasons 2008/09-2010/11) and pandemic influenza (season 2009/10), to identify predictors of and barriers to pandemic vaccine uptake and whether the controversial discussions on pandemic vaccination has had a negative impact on seasonal influenza vaccine uptake in Germany.

**Methods:**

We analysed data from the ‘German Health Update’ (GEDA10) telephone survey (n=22,050) and a smaller GEDA10-follow-up survey (n=2,493), which were both representative of the general population aged ≥18 years living in Germany.

**Results:**

Overall only 8.8% of the adult population in Germany received a vaccination against pandemic influenza. High socioeconomic status, having received a seasonal influenza shot in the previous season, and belonging to a target group for seasonal influenza vaccination were independently associated with the uptake of pandemic vaccines. The main reasons for not receiving a pandemic vaccination were ‘fear of side effects’ and the opinion that ‘vaccination was not necessary’. Seasonal influenza vaccine uptake in the pre-pandemic season 2008/09 was 52.8% among persons aged ≥60 years; 30.5% among HCW, and 43.3% among chronically ill persons. A decrease in vaccination coverage was observed across all target groups in the first post-pandemic season 2010/11 (50.6%, 25.8%, and 41.0% vaccination coverage, respectively).

**Conclusions:**

Seasonal influenza vaccination coverage in Germany remains in all target groups below 75%, which is a declared goal of the European Union. Our results suggest that controversial public discussions about safety and the benefits of pandemic influenza vaccination may have contributed to both a very low uptake of pandemic vaccines and a decreased uptake of seasonal influenza vaccines in the first post-pandemic season. In the upcoming years, the uptake of seasonal influenza vaccines should be carefully monitored in all target groups to identify if this trend continues and to guide public health authorities in developing more effective vaccination and communication strategies for seasonal influenza vaccination.

## Background

In Germany, annual influenza epidemics usually occur during the winter months December to March. In the last decade, an estimated zero to 19,000 excess deaths per year were attributable to influenza virus infections [1]. Moreover, approximately one to six million influenza-related excess physician consultations per season were estimated for Germany [2]. Severe influenza virus infections or influenza-related complications typically occur in the very young and elderly population as well as in persons with underlying chronic medical conditions.

Annual vaccination has proven to be an effective method to reduce the burden of influenza disease [3]. In Germany, vaccination against seasonal influenza is recommended by the Standing Committee on Vaccination (STIKO) for individuals who have either an increased risk to develop severe influenza disease (i.e. persons aged ≥60 years, pregnant women, and persons with certain chronic medical conditions) or who are likely to transmit the virus to vulnerable groups (e.g. health care workers (HCW)) [4]. Vaccination is free of charge for the target groups in Germany. During the influenza pandemic 2009/10, STIKO additionally recommended vaccination with a monovalent vaccine against the pandemic influenza virus strain A(H1N1)pdm09 for the whole population. Due to expected limitations in vaccine supplies at the beginning of the vaccination campaign, STIKO defined and ranked priority groups for the pandemic vaccination: 1) HCW, 2) persons with underlying chronic conditions, 3) pregnant women, 4) household contacts of vulnerable persons, 5) all other persons aged 6 months to 24 years, 6) all other persons aged 25–59 years, 7) all other person aged 60 years and above [5]. The pandemic vaccination campaign started in Germany on 26 October 2009 [6]. The AS03-adjuvanted monovalent vaccine Pandemrix® was almost exclusively used and available in sufficient quantities [7].

During the pandemic, vaccination against A(H1N1)pdm09 was subject to controversial discussions, not only in Germany but also in many other countries worldwide [7-9]. Main topics of the debate in the media and among experts and ‘self-proclaimed experts’ were vaccine safety, effectiveness, and concern that there were too little data on the new vaccines or vaccine ingredients (especially new adjuvants) available. Moreover, the general necessity of vaccination in view of the relative mildness of the pandemic influenza disease was called into question [7,9-11]. As a result, compliance with the national recommendations for pandemic vaccination was very poor in Germany. Since Germany has no central immunization registry, information on vaccination coverage (and factors influencing coverage) is only available from telephone and household surveys [12-15]. According to the results of thirteen consecutive cross-sectional telephone surveys (total n=13,010) conducted during the pandemic, only 8.1% of the general population aged ≥14 years living in Germany received a vaccine against pandemic influenza [6].

To develop target group specific communication strategies and to enhance compliance with the official recommendations it is important to monitor vaccine uptake in each of the target groups and to understand factors that influence uptake. This applies not only for annual influenza vaccination campaigns but also for the planning of future vaccination campaigns during a pandemic. The influence of the 2009/2010 pandemic situation on seasonal influenza vaccine uptake in Germany in the post-pandemic seasons has so far not been investigated. For this purpose, we utilized data from the large (~22,000 respondents) ‘German Health Update 2010’ (GEDA10) telephone survey and a smaller GEDA10 follow-up survey (~2,500 respondents). The objectives of our study were (1) to assess seasonal influenza vaccination coverage for seasons 2008/09 to 2010/11, (2) to assess pandemic influenza vaccination coverage for season 2009/10, (3) to identify predictors of and barriers to pandemic vaccine uptake, and (4) to detect a potential influence of the pandemic situation on seasonal influenza vaccine uptake in the first post-pandemic season (2010/11).

## Methods

### Study population and survey design

The GEDA survey design has been described previously [12,13,16]. In brief, GEDA is a large annual telephone survey which is conducted by the Robert Koch Institute (RKI) as a part of Germany’s national health monitoring. The study population consists of persons ≥18 years of age who are living in a private household in Germany, have sufficient knowledge of the German language, and can be contacted via landline telephone. The GEDA study protocol was approved by Germany’s federal and regional data-protection commissioners. All data were collected and analysed in an anonymous manner.

In this study we present data from GEDA10 which was conducted between 22 September 2009 and 10 July 2010. Since the annual GEDA survey was not conducted in 2010/2011, we conducted a follow-up interview among a subsample of 2.493 GEDA10 respondents (from now on referred to as the GEDA follow-up survey) from 1 April to 2 July 2011 to assess seasonal influenza vaccine uptake for the post pandemic season 2010/11. Based on a sample size calculation, 385 subjects were needed to estimate a prevalence of 50% (“worst case scenario”) vaccine uptake with a confidence interval of +/− 5%. Our sample size of ~2,400 subjects for the follow-up survey was based on the premise to estimate a prevalence (i.e. vaccination coverage) of 50% for up to six subgroups (=cells; 6*385≈2400).

Since both surveys were conducted by RKI, the ownership of the data lies with RKI and we did not have to obtain permission to use the data for this study. Similar to the previous GEDA survey (GEDA09), a public use file of the GEDA10 dataset will be provided soon. Data from the GEDA follow-up survey will not be openly available.

To control for possible selection biases, weighting factors for the GEDA10 sample were constructed by taking age, sex, educational status, geographical region, and household size into consideration. Potential participants of the follow-up survey were sampled disproportionally to their weighting factors in GEDA10. We applied this method to avoid that groups which were already underrepresented in GEDA10 become underrepresented again at the sampling stage in the follow-up sample and thus to prevent further bias. Weighting factors for the follow-up survey were constructed in a first step on the basis of the values calculated for GEDA10 (for age, sex, education, adipositas, smoking status, subjective health, physical activity, employment status), and, in a second step, on population data gathered in the Microcensus 2008 [17], taking geographical region, age, sex, and educational status into account.

Information on seasonal influenza vaccination status for season 2008/2009 was collected from all participants of GEDA10. Between 1 January 2010 and 10 July 2010 respondents were additionally asked to provide information on seasonal and pandemic influenza vaccination status for season 2009/10. Unvaccinated survey participants of GEDA10 were additionally asked to state their reasons for not receiving a pandemic influenza vaccination. Information on seasonal vaccination status for season 2010/11 was collected from all respondents of the GEDA follow-up survey. In the follow-up survey, respondents were additionally asked by whom they were vaccinated (general practitioner/other physician in private practice/occupational physician/other) and in which month they received the vaccination against seasonal influenza in season 2010/11. One should note that GEDA10 does not cover the paediatric population (aged 0–17 years) for which pandemic vaccination was also recommended in Germany. Information on vaccination coverage in this particular age-group was therefore not available from this data source.

We calculated the response for GEDA10 by using Response Rate 3 as defined by the American Association for Public Opinion Research (AAPOR) [18]. Response Rate 3 is the proportion of the number of complete interviews divided by the number of interviews plus the number of non-interviews (refusal and break-off plus non-contacts plus others) plus cases of unknown eligibility. For cases of unknown eligibility Response Rate 3 estimates what proportion of cases of unknown eligibility is actually eligible. This estimation is based on the proportion of eligible households among all numbers for which a definitive determination of status was obtained (hence a very conservative estimate). We additionally calculated the cooperation rate at respondent level, which is defined as the proportion of all respondents interviewed of all respondents ever contacted [18]. Since the GEDA follow-up survey was not a random digit dialling study (as GEDA10), we reported the minimal response rate (Response Rate 1 as defined by AAPOR, [18]) for the follow-up survey.

### Definition of variables

Socio-economic status levels were created as described by Lampert and Kroll on the basis of self-reported educational, income, and professional status of survey respondents [19]. In accordance with the STIKO-recommendations [4], persons were classified into the target groups for seasonal influenza vaccination in our study if they reported (1) to be ≥60 years of age, (2) to have at least one underlying chronic disease (defined here as having a chronic underlying respiratory, cardiovascular, liver, or renal disease, cancer, or diabetes), or (3) to work as HCW. Since female respondents of child-bearing age were not asked whether they had been pregnant during the last influenza season, it was not possible to include pregnant women as target group in our analysis. The geographic region category ‘Western Federal States’ (WFS) comprised the federal states Schleswig-Holstein, Bremen, Hamburg, Lower Saxony, Hesse, Rhineland-Palatinate, Saarland, North Rhine-Westphalia, Baden-Württemberg and Bavaria; ‘Eastern Federal States’ (EFS) comprised Mecklenburg-Vorpommern, Brandenburg, Berlin, Saxony-Anhalt, Thuringia and Saxony.

### Statistical analysis

Data analysis was performed using PASW 18.0 for Windows (SPSS Inc., Chicago, USA). Proportions were calculated by using procedures for the analysis of complex samples. Univariate analyses were conducted to determine associations between pandemic influenza vaccine uptake and socio-demographic, health-related and professional factors. A p-value ≤0.05 was considered to indicate a statistically significant difference. Odds ratios (OR) and 95% confidence intervals (CI) were calculated as appropriate. Multivariable analysis was performed by entering variables potentially associated with vaccine uptake (p-value <0.2 in univariate analysis) into a multivariable logistic regression model in a first step, followed by step-wise backward removal of variables with a p-value >0.05 to produce a final model. Interaction terms were included to account for effect modification between independent variables.

## Results

### Sample characteristics

In total, 22,050 telephone interviews were conducted during the study period of GEDA10 and 2,493 participants were re-interviewed for the follow-up survey. An overview of the survey populations is given in Table 1. The median age was 48.0 years (range 18–99 years) in GEDA10 and 49.7 years (range 19–96 years) in the follow-up survey. Response Rate 3 was 28.9% in GEDA10; the cooperation rate at respondent level was 55.8%. Response Rate 1 was 75.0% in the follow up survey.

**Table 1:** **Characteristics of participants in the** ‘**German Health Update Survey**’ (**GEDA10**) **and the GEDA10 follow**-**up survey**

	**GEDA10 *September 2009*** – ***July 2010***		**Follow**-**up survey *April*** – ***July 2011***	
	n^*^	%^*^	n^*^	%^*^
**Total**	22,050	100	2,493	100
**Sex**				
female	11,347	51.5	1,287	51.6
male	10,703	48.5	1,206	48.4
**Age group**				
18-39 years	7,145	32.4	761	30.5
40-59 years	8,142	36.9	937	37.6
≥60 years	6,763	30.7	795	31.9
**Place of residence**				
Western Federal States	17,433	79.1	1,968	79.0
Eastern Federal States	4,617	20.9	525	21.0
**Underlying chronic disease**				
yes	7,260	32.9	972	39.0
no	14,790	67.1	1,521	61.0
**Health care worker**				
yes	1,117	5.1	225	9.0
no	20,933	94.9	2,268	91.0

*Weighted data.

### Seasonal influenza vaccination coverage

**Figure 1:**
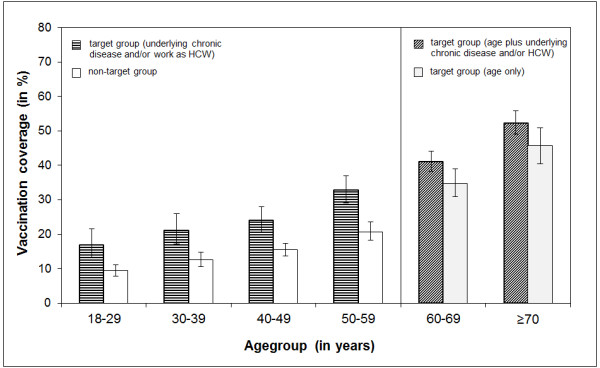
**Seasonal vaccination coverage by age group and target group**, **season 2009/10**.

Information on seasonal influenza vaccination status was available for over 99.8% in each of the study samples for the three seasons under investigation (seasons 2008/09-2010/11). Vaccination coverage for the three seasons by sex, age group, place of residence, and target group is presented in Table 2. To allow comparability with international studies, seasonal influenza vaccine uptake among ≥65 year-olds was additionally calculated and revealed a coverage of 56.1% (95% CI: 54.0-58.2) in season 2008/09, 50.2% (95% CI: 47.5-52.9) in season 2009/10, and 54.2% (95% CI: 48.4-59.8) in season 2010/11. For influenza season 2009/10, vaccination coverage was calculated by age-groups in decades (Figure 1). Vaccine uptake in both the target population for seasonal influenza vaccination (defined here as persons who have an underlying chronic disease, or work as HCW) and non-target population increased with age and was highest in persons ≥70 years. The vast majority (97.8%) of vaccinated persons had received their influenza vaccination for season 2010/11 by the end of December 2010.

**Table 2:** **Seasonal influenza vaccine uptake by sex**, **age group**, **place of residence and target group**, **seasons 2008**/**09** − **2010**/**11**, **Germany**

	**Season 2008**/**09**	**Season 2009**/**10**	**Season 2010**/**11**
	***n***=***22***,*009 *% (95% CI)^*^	***n***=***13***,*040 *% (95% CI)^*^	***n***=***2***,*492 *% (95% CI)^*^
**Total**	29.8 (29.0-30.6)	26.6 (25.6-27.6)	28.3 (26.0-30.6)
**Sex**			
female	30.8 (29.7-31.9)	27.2 (25.8-28.6)	28.2 (25.3-31.3)
male	28.8 (27.6-30.0)	26.0 (24.5-27.5)	28.3 (25.0-32.0)
**Age group**			
18-39 years	14.8 (13.8-15.9)	12.8 (11.6-14.1)	13.3 (10.7-16.6)
40-59 years	23.9 (22.8-25.0)	21.2 (19.8-22.6)	21.5 (18.6-24.7)
≥60 years	52.8 (51.0-54.5)	47.5 (45.2-49.8)	50.6 (45.7-55.5)
**Place of residence**			
Western Federal States	26.7 (25.8-27.6)	24.0 (22.9-25.1)	25.8 (23.3-28.4)
Eastern Federal States	41.5 (39.6-43.5)	36.7 (34.3-39.2)	37.7 (32.6-43.0)
**Target group**			
health care workers	30.5 (27.6-33.6)	27.3 (23.6-31.3)	25.8 (20.4-32.1)
chronically ill persons	43.3 (41.7-44.9)	39.8 (37.8-41.9)	41.0 (36.9-45.3)
non-target group	16.6 (15.8-17.5)	14.2 (13.2-15.3)	14.8 (12.5-17.5)

*Weighted data.

**Figure 2:**
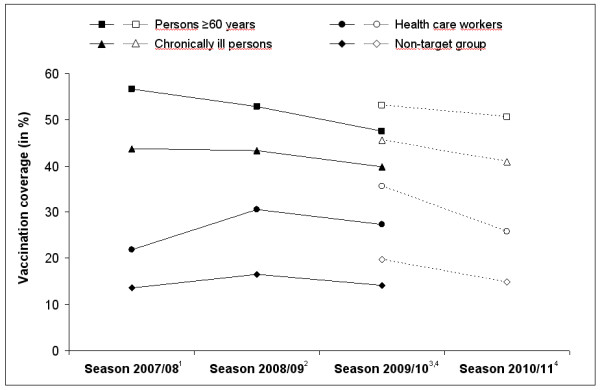
**Trends in seasonal vaccine uptake in target groups in Germany for seasons 2007/08−2010/11 according to GEDA09 and GEDA10(filled symbols)and a follow-up sample of GEDA10(empty symbols).**^**1**^ Data source: GEDA09 (n=15,552) [12]; ^2^ Data source: GEDA10 (n=22,009); ^3^ Data source: GEDA10 (n=13,040); ^4^ Data source: GEDA follow-up survey (n=2,492).

Figure 2 shows trends in seasonal influenza vaccine uptake among the three different target groups and the non-target group for four consecutive seasons (2007/08 to 2010/11; results for season 2007/08 according to a previously published analysis of GEDA 2009 data [12]). While vaccination coverage slightly decreased in persons aged ≥60 years and in persons with underlying chronic diseases between seasons 2007/08 and 2009/10, there was an increase in vaccine uptake among HCWs from season 2007/08 to 2008/09. In all subgroups under investigation, vaccine uptake for season 2009/10 was higher in the follow-up sample (empty symbols) compared to the GEDA10 sample (filled symbols). Considering only the results from the follow-up survey (Figure 2), a significant decrease in seasonal influenza vaccination coverage between seasons 2009/10 and 2010/11 was observed for persons with underlying chronic conditions (p=0.04), HCWs (p=0.03), and persons not targeted for seasonal influenza vaccination (p<0.01). For persons ≥60 years of age there was also a decrease, but without reaching statistical significance. In season 2010/11, 83.4% of respondents who received a seasonal influenza shot were vaccinated by their general practitioner, 4.7% by another physician in private practice (e.g. gynaecologist, paediatrician), 9.4% by an occupational physician, 0.4% by a hospital physician, and 2.1% by any other physician. Of those who were vaccinated by a general practitioner or by an occupational physician, 18.4% and 59.4% did not belong to a target group, respectively.

### Pandemic influenza vaccination coverage

Information on pandemic influenza vaccine uptake was available for 99.9% of the respective study population in GEDA 10 (n=13,048). Vaccination coverage by sex, age group, place of residence, socio-economic status and different target groups for seasonal influenza vaccination is presented in Table 3. In total, 8.8% (95% CI: 8.2-9.5) of the general adult population in Germany received a vaccination against pandemic influenza. With 11.2% (95% CI: 10.2-12.3) pandemic vaccine uptake was significantly higher in persons belonging to the target group for seasonal influenza vaccination as compared to the non-seasonal influenza target group (6.4%; 95% CI: 5.7-7.1; p<0.001).

**Table 3:** Determinants of pandemic influenza vaccine uptake, Germany, season 2009/10

	**n**^**#**^	**A(H1N1)pdm09 vaccination coverage; % (95%CI)**^**#**^	**Odds Ratio (95%CI)**^**#**^	
			**univariate**	**multivariable**
**Total**	13,032	8.8 (8.2-9.5)	-	-
**Sex**				
female	6,730	7.8 (7.1-8.7)	1^ref^	1^ref^
male	6,302	9.9 (8.9-10.9)	1.29 (1.14-1.45)^**^	1.36 (1.15-1.61)^**^
**Vaccinated against seasonal influenza in season 2008/09**				
*for agegroup 18*–*59 years*				
no	7,218	4.4 (3.8-4.9)	1^ref^	1^ref^
yes	1,784	22.9 (20.7-25.3)	6.54 (5.43-7.87)^**^	5.98 (4.96-7.20)^**^
*for agegroup* ≥*60 years*				
no	1,925	1.9 (1.3-2.8)	1^ref^	1^ref^
yes	2,078	18.5 (16.1-21.1)	11.60 (7.51-17.01)^**^	4.76 (1.60-14.18)^**^
**Place of residence**				
WFS	10,338	8.9 (8.2-9.6)	1^ref^	n.s.
EFS	2,694	8.7 (7.3-10.3)	0.98 (0.84-1.14)	
**Socioeconomic Status**				
low	2,524	7.6 (6.1-9.5)	1^ref^	1^ref^
medium	7,777	8.0 (7.3-8.9)	1.06 (0.89-1.25)	1.01 (0.77-1.33)
high	2,688	12.2 (11.1-13.4)	1.69 (1.40-2.03)^**^	1.61 (1.23-2.11)^**^
**Health care workers**				
no	687	8.4 (7.8-9.1)	1^ref^	1^ref^
yes	12,345	16.5 (13.9-19.6)	2.16 (1.75-2.67)^**^	2.30 (1.78-2.96)^**^
**Underlying chronic disease**				
no	8,786	7.2 (6.6-7.9)	1^ref^	1^ref^
yes	4,246	12.2 (10.9-13.6)	1.79 (1.58-2.02)^**^	1.42 (1.19-1.69)^**^

^#^weighted data; * p<0.05; ** p<0.001; ^ref^=reference category; n.s.= not significant; WFS=Western federal states, EFS=Eastern federal states.

p-value for interaction between agegroup*seasonal influenza vaccination status: 0.011.

The most frequently reported reasons for not receiving the vaccination were (1) ‘fear of side effects of pandemic vaccines’ (stated by 37.2%; 95% CI: 36.1-38.3), (2) ‘pandemic vaccination is not necessary’ (33.8%; 95% CI: 32.7-34.9), (3) ‘pandemic vaccination not officially recommended for me’ (16.6%; 95% CI: 15.8-17.5), and (4) ‘reject vaccinations in general’ (8.5%; 95% CI: 7.8-9.2).

### Factors associated with pandemic influenza vaccine uptake

Results of univariate and multivariable analysis of factors potentially associated with pandemic influenza vaccine uptake are shown in Table 3. Having received a seasonal influenza vaccination in the previous season (season 2008/09) was the strongest independent predictor of pandemic influenza vaccination. However, this effect differed by age group and we therefore included an interaction term in the final model. Additionally, working as HCW, having a chronic disease, high socioeconomic status, and being male were significantly associated with higher uptake in multivariable analysis.

## Discussion

The aim of this study was to assess the uptake of seasonal influenza vaccines in specific target groups for seasons 2008/09 and 2009/10, as well as for pandemic influenza vaccines during the pandemic season 2009/10 in Germany in the total adult population by using data from a large population-representative telephone survey. By using data from a smaller follow-up survey, our study moreover provides the only so far available data on seasonal influenza vaccination coverage in Germany for the post-pandemic season 2010/11. Overall, only 8.8% of the adult population in Germany followed the official recommendation and received a vaccination against pandemic influenza in season 2009/10. The follow-up survey revealed a decrease in seasonal influenza vaccine uptake in the first post-pandemic season across all target groups when compared to the pre-pandemic season 2008/09, most prominent among HCW. With an average coverage of 50% in the elderly, 41% in the chronically ill, and 28% in HCW in seasons 2008/09 to 2010/11, the EU goal of reaching a seasonal influenza vaccination coverage of at least 75% in the target groups [20] has not yet been achieved in Germany.

Having received a seasonal influenza shot in the pre-pandemic season was the strongest predictor for receiving pandemic influenza vaccination in our study. The high correlation between seasonal and pandemic influenza vaccine uptake highlights the significance of habitual behaviour with regard to influenza vaccination decisions. In addition and independent from this factor, persons belonging to at least one of the recommended target groups for seasonal influenza vaccination were significantly more likely to receive a pandemic influenza vaccination than persons not belonging to a target group. Our results are broadly in line with the findings of a prospective monitoring survey on pandemic influenza vaccination in Germany [6] and two reviews investigating determinants of pandemic vaccine uptake [21,22]. Prior seasonal vaccination was not only found to be positively associated with the intention to receive the pandemic vaccination among adults in several industrialized countries (e.g. in the UK [23], France [24], Australia [25], and the US [26]) but also with the actual receipt of the vaccination (e.g. [6,27,28]). When developing vaccination strategies for future pandemic situations one should therefore consider targeted strategies for enhancing coverage among those who do not fall within the target groups for seasonal influenza vaccination and thus do not regularly receive a seasonal influenza shot. A further opportunity to enhance compliance with national recommendations and therefore vaccination coverage in a future pandemic situation could be to increase seasonal vaccine uptake in the target groups. However, major reasons for not being vaccinated were the perception that vaccination was not necessary or not safe. It can be assumed that both reasons will not be barriers to high pandemic vaccine uptake in a future pandemic setting if the mortality is much higher than during the 2009/10 pandemic.

A higher uptake of seasonal influenza vaccines in season 2009/10 was observed in the follow-up survey population when compared to the total GEDA10 study population in 2009/10. It is therefore very likely that the point estimates for seasonal influenza vaccination coverage for the 2010/11 season, which were based on data from the same follow-up survey, were also overestimated. Taking into consideration that acceptance of seasonal influenza vaccination was higher in the follow-up survey population, our study results suggest that seasonal influenza vaccine uptake in the recommended target groups in Germany has decreased in the post-pandemic season 2010/11, not only in comparison to season 2008/09 but also to the pandemic season 2009/10 (compare Figure 2, empty symbols). Hence, our findings are discordant with observations made in several other industrialised countries. For instance, seasonal influenza vaccination coverage in the UK remained stable between seasons 2009/10 and 2010/11 among at risk persons under 65 years of age (51.6% vs. 50.4% vaccination coverage) as well as among persons aged ≥65 years (72.4% vs. 72.8%) [29]. Among high-risk persons aged 18–64 years living in the US, seasonal influenza vaccine uptake was 46.2% in the pandemic and 46.7% in the post-pandemic season [30]. However, in both countries acceptance and uptake of pandemic influenza vaccination was higher as compared to Germany (UK: 37.6% vaccination coverage in clinical risk groups [29]; US: 41.2% among all persons aged ≥6 month [31]). In France, despite the poor uptake of pandemic influenza vaccines (11.1%), an increase in seasonal influenza vaccine uptake was observed in the post-pandemic season among persons aged ≥65 years with underlying chronic conditions (62.6% in season 2009/10 [27] vs. 71.0% in season 2010/11 [32]). In the upcoming years, the uptake of seasonal influenza vaccines should be carefully monitored in Germany in all target groups to identify if this trend continues. Especially the strong decrease in vaccination coverage among HCW is of concern, and communication activities should be strengthened especially for this target group not only to achieve individual protection of this target group but also to protect vulnerable patients managed by HCW.

In our study ‘fear of side effects’ was found to be the most frequently stated reason for rejecting pandemic vaccination, thereby confirming findings of 13 smaller consecutive surveys carried out during the pandemic in Germany [33]. Conversely, believing that the pandemic vaccine is safe was significantly associated with the receipt of the pandemic vaccine in many countries worldwide [21]. Appropriate addressing of vaccine safety concerns by public health authorities may be an important factor to maintain public trust in national vaccination recommendations and beyond that to enhance vaccine uptake in future pandemic situations [22,33].

In our study, 8.5% of those who did not receive a pandemic influenza vaccination stated that they reject vaccinations in general. This translates into a proportion of 7.7% for the total study population. Little is known about the exact proportion of vaccination opponents among the general adult population in Germany. In a recent survey performed by the German Federal Centre for Health Education (BZgA) among 3,002 parents of children aged 0–13 years, 35% stated that they reject particular vaccinations for their children, but only 1% of parents reject vaccinations in general [34]. Since our study did not focus on the general rejection of vaccinations in the population, we did not ask further detailed questions related to this topic to verify this attitude and the underlying reasons. Therefore, this figure must be interpreted with caution.

Our study has some limitations that need to be acknowledged. Calculation of influenza vaccination coverage was based on self-reported vaccination status and may therefore be prone to recall problems. However, it was shown in several studies that self-report of influenza vaccination status has an adequate degree of validity [35,36]. Furthermore, the response rate in GEDA10 was comparatively low at 29%. However, it should be noted that the chosen method of calculating the response rate (namely Response Rate 3 as defined by AAPOR [18]) is a very conservative approach and that our response rate is comparable to studies using the same approach (e.g. CDC-Behavioral Risk Factor Surveillance Rates Report [37]). Finally, it cannot be ruled out that other reasons than the controversial discussions on the pandemic vaccination have also contributed to the observed drop in seasonal influenza vaccination coverage in 2010/11.

## Conclusion

In conclusion, poor compliance with official vaccination recommendation resulting in low uptake of pandemic influenza vaccines during the pandemic season 2009/10 suggests that public communication strategies and vaccination campaigns during the influenza A(H1N1)pdm09 pandemic in Germany were not successful. In addition, our results raise concerns that controversial discussions about the safety and necessity of pandemic influenza vaccines may have contributed to decreased seasonal influenza vaccine uptake in the first post-pandemic season. It is therefore crucial to develop concerted communication strategies based on the lessons learned from the 2009/10 influenza pandemic and to include them in the national pandemic preparedness plan. This should be done, not only with respect to a competent handling of pandemic situations but also to avoid a decrease in the acceptance of vaccinations in general. In this respect, communication strategies and different modes of communication to specific target groups should be evaluated and implemented already in non-crisis situations, to be enhanced during a pandemic influenza situation or other public health crisis. This is of particular importance, since seasonal influenza vaccine uptake in the recommended target groups in Germany stagnated at a low level since 2005 [38] and does by far not meet the EU goal of 75% [20]. Further studies should be conducted to monitor the trends of seasonal influenza vaccine uptake in Germany in the specific target groups including pregnant women (which is a target group for seasonal influenza vaccination since 2010) and to precisely identify barriers to influenza vaccination in the upcoming years which might differ from the pandemic and the first post-pandemic season. This information would be crucial to guide public health authorities in developing more effective communication strategies for seasonal influenza vaccination tailored to specific target groups.

## Competing interests

The authors have declared no conflict of interest.

## Authors’ contributions

All authors made substantial contributions to the study. SM was involved in the development of the GEDA10 study design and contributed to the Methods section. MMB, DW, and OW developed the study design of the GEDA10 follow-up survey. MMB analysed the data in consultation with DW, GF, OW and GK. MMB wrote the draft version of the manuscript. All authors have read, carefully reviewed and approved the final version of the manuscript.

## Pre-publication history

The pre-publication history for this paper can be accessed here:

http://www.biomedcentral.com/1471-2458/12/938/prepub
